# Craniofacial features of children with spinal deformities

**DOI:** 10.1186/1471-2474-9-169

**Published:** 2008-12-22

**Authors:** Emil Segatto, Carsten Lippold, András Végh

**Affiliations:** 1 Department of Orofacial Orthopedics and Orthodontics, Heim Pál Children's Hospital, Zoltán u 18, 1054 Budapest, Hungary; 2 Department of Orthodontics, University of Münster, Münster, Waldeyerstr. 30, 48149 Münster, Germany

## Abstract

**Background:**

The objective of this epidemiological study is to map the dentofacial anomalies that can be correlated to the two most frequent spinal diseases responsible for postural abnormalities and that can be clinically identified by the orthodontic examination.

**Methods:**

Twenty-three children with Scheuermann's disease participated in the study (mean age: 14Y8M; SD: 1Y8M), 28 with Scoliosis (mean age: 14Y7M; SD: 2Y3M) and a control group of 68 orthopedically healthy children (mean age: 14Y8M; SD: 0Y11M). Standardized orthodontic screening protocols were used to map the occlusal relations in the sagittal, vertical, and transversal dimensions, space relations of the maxillary and mandibular frontal segment, and the TMJ status and function. The examinations for the children with orthopedic disorders were supplemented by the evaluation of routine orthodontic radiograms – lateral cephalograms and panoramic X-rays.

**Results:**

The majority of the dentofacial features examined revealed more and greater abnormalities among patients in the Scheuermann's disease group than in the scoliosis group. In the latter group the proportion of the TMJ symptoms and the consecutive functional deviations were greater. When comparing the values of the two spinal-disorder groups and the control group, statistically significant differences (*p *< .05) occurred for the following measurements: frequency of unilateral Cl.II. molar occlusion, overjet and extreme overjet mean value (Scheuermann's disease group), as well as the frequency of TMJ pathological symptoms (scoliosis group). The evaluation of the panoramic X-rays showed significant differences among the mandibular measurements of the two spinal-disorder groups. Within the framework of the evaluation of the cephalograms significant differences (*p *< .05) were found only in the case of dental relations. However, several values differed significantly from the Ricketts' norms, none of the indices strictly characterized any of the groups with spinal disorders.

**Conclusion:**

The more extended treatment of the malocclusions closely correlated to postural disorders draws attention to the indicators of a higher frequency and severity occurring in the case of the dentofacial deviations in the patients of the MSCH group who had previously been less examined.

## Background

The literature exploring the reasons for the development of malocclusions concentrates very much on the connections to the different postural anomalies. Those postural anomalies are mainly highlighted which could contribute to the development of the different dentofacial anomalies and later to the sustenance thereof by their chronic influence on the head posture [1,2]. Thus, those orthopedic disorders become very important that mainly become manifest in pathological curves of the spine [3]. According to literature sources, the frequency of various malocclusions in the orthopedic patient group amounts to 83–87% [4].

The role of the head posture that is tilted forwards and backwards evolved as a consequence of the pathological curves of the sagittal plane (kyphosis and lordosis) which were mainly examined within the framework of the study of the development of the sagittal and vertical jaw anomalies [5-10]. In their opinion, the scoliotic curves occurred in the frontal plane – through the head posture that is tilted sideways – and played an important role in the development of the different dentofacial asymmetries [9]. The alteration of the head posture may lead to the development of TMJ dysfunction and TMJ structural deformity [11]. Because of their similar epidemiologic characteristics – incidence, stability of the developed deformations, time of appearance – the two spinal diseases which are considered as the most appropriate in terms of the examinations are the Scheuermann's disease (or kyphosis dorsalis juvenilis) and the idiopathic scoliosis [12]. The occurrence of the Scheuermann's disease varies between 0.4 and 11%. The pathological kyphotic curvature which is liable for the head posture tilted forward and hunchbacked appearance is primarily pronounced and stabilized in the dorsal spinal region. Its development begins during pre-puberty and is not characterized by gender deflection [13-15]. The frequency of the idiopathic scoliosis alters between 11.9 and 16.2%. The main curvature may be localized in any part of the vertebral column, the most common being the right convex dorsalis scoliosis which – in the non-compensated forms – induces the head posture to be tilted to the left. Its development starts during the pre-puberty, and – as regards the gender distribution – it occurs 7–10 times more often in girls than in boys [13,16,17]. The conservative treatment in the early stage of both spinal diseases consists in posture strengthening and the improvement of the muscle tone by physical therapy, while in late, severe stages of the disease, it is necessary to wear a corset. The reason for this is the deformed vertebra's malformation stage in the case of Scheuermann's disease, while in patients with scoliosis the Cobb value of more than 20 degrees warrants the corset [16,18]. However, the severe deterioration cannot be improved by the conservative treatments but only with the help of surgical interventions.

We performed the orthodontic examination on children suffering from Scheuermann's kyphosis and from idiopathic scoliosis in order to collect data for further related studies. Based on the results we wanted to get a more accurate image of the craniofacial characteristics, the functional habits and the occlusal patterns of patient groups with spinal deformities in the sagittal and frontal plane. The patients participating in the epidemiological survey received detailed information on the examinations in advance; their parents provided their written consent to the performance thereof. The examinations complied with the requirements of the local Ethics Committee.

## Methods

### a) Subjects

**Figure 1:**
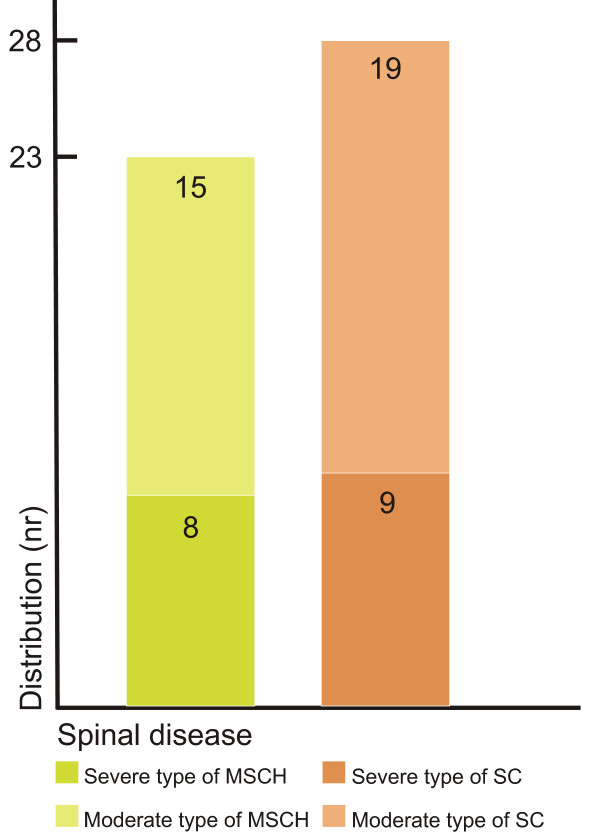
**Distribution of the MSCH and SC groups based on severity**.

For the epidemiological study, the Orthopedic Department referred 65 patients who had been recently diagnosed with Scheuermann's disease and idiopathic scoliosis. Fourteen of these patients actually had a history of previous orthodontic treatment; hence they were excluded from the study. The remaining patients consisted of 51 children, 23 of whom formed the group with Scheuermann's disease (MSCH) and 28 the group with scoliosis (SC). Due to the differences in gender distribution in the two orthopedic groups, the data of the girls and boys were evaluated together. Both groups were divided into two subgroups in order to assess the relationship between the extent of the dentofacial deviations and the severity of the orthopedic disease. The classification was based on the severity determining the therapy used for the orthopedic malformation. In the MSCH group those wearing indicated corset on the basis of deformed vertebra's malformation stage were put into the severe group. In the SC group the indication of the corset was represented by the values of Cobb angle > 20° measured at the level of main curvature, so these children belonged to the severe group. In the MSCH group eight children and in SC group nine children were wearing corset because of the severity of their orthopedic malformation. In the case of both patient groups the moderate subgroups consisted of children with malformations requiring no constant posture correction – namely wearing corset [Figure 1]. For the more adequate ratability of the results the survey was extended to include a group of 93 subjects considered healthy from orthopedic point of view. Besides the clarified negative orthopedic anamnesis the selection criteria of this group were: similar age, no previous orthodontic treatment, as well as no missing teeth, carious lesions, or pathologic periodontal status. Sixty-eight individuals met the above mentioned criteria; they formed the control (CTRL) group.

### b) Equipment

For the examination of the selected children the standardized orthodontic protocol was used, including clinical examination and orthodontic impressions. In order to get an exact evaluation the results were tabulated on the WHO broadsheet used for epidemiological studies. The clinical examinations were supplemented for the patients with spinal diseases by the routine lateral cephalogram and the panoramic X-ray pictures (Orthophos DS Ceph, Siemens, Bensheim, Deutschland; Focus-CCD sensor distance 1.2 m; tube values: 60–90 kV, 9–16 mA).

### c) Cephalometric examination

**Figure 2:**
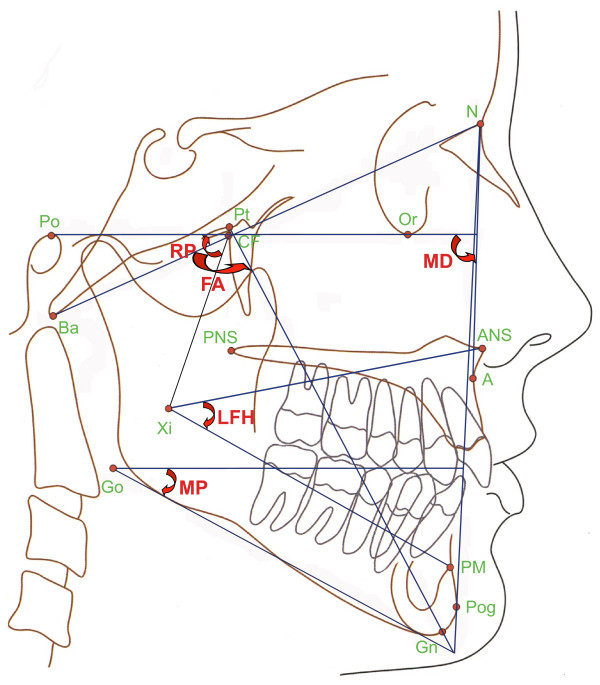
**Cephalometric analysis**.

The evaluation of the cephalograms was done according to Ricketts by two examiners using FR-Win orthodontic analyzing software (Computer Konkret, Falkenstein, Germany). In order to maintain anonymity during the evaluations, the children's names were blinded. The methodological error was determined by means of the Dahlberg formula [19] (mean error ratio SE^2 ^= d^2^/2n, where d = difference between the measurements taken by two examiners; n = sample size). The following measurements are considered to be relevant for the study: maxillary depth (MD), ramus position (RP), facial axis (FA), lower facial height (LFH) and mandibular plane (MP) [Figure 2].

The panoramic X-ray evaluation was done on the basis of the measuring method by Habets et al [20] under similar conditions. With the help of the distances measured on the two mandibular ramuses (codylar/CH/, ramal/RH/, and condyle plus ramal/CH+RH/height) determined asymmetry indices served for mapping the skeletal asymmetry present at this bone level. For all the three distances the determination of the asymmetry index characteristic to the given length occurred with the help of the formula [(R-L)/(R+L)] × 100, by using the values measured on the right (R) and on the left (L) ramuses.

### d) Orthodontic examination

The examination of the orthodontic characteristics are grouped around three issues:

• Mapping of the occlusal and skeletal characteristics in the sagittal, transversal and vertical direction

• Examination of the jaw space relations

• Examination of temporomandibular joint

The measurement of the occlusal characteristics in the sagittal plane was based on the determination of the molar relation (Angle classification), and on the overjet of the incisors. This was supplemented by sagittal features like the maxillary depth (MD) and ramus position (RP) taken from the cephalometric analysis. The presence of the transversal abnormalities was measured by the registration of the crossbite in the molar region, of the upper and lower midline in relation to the facial midline and of the midline deviation measured between upper and lower dental midlines. In order to study the vertical abnormalities the lateral open bite was registered and the overbite of the incisors was measured. The facial axis (FA), the lower facial height (LFH) and the mandibular plane (MP) are the vertical skeletal characteristics derived from the cephalometric evaluation. For the examination of the jaw space relations the crowding and the presence of spaces in the incisor region were recorded. In the pursuance of the profound examination of the TMJ the abnormal symptoms (clicking of the joint, pain, limited mouth opening), the mentum deviation during mouth opening, and the rate of the lateral movement were marked and measured.

### e) Data analysis

The sheets were evaluated and the values statistically analyzed by means of SPSS (Lead Tech Chicago, Ill, USA) with the significance level in all tests being determined to be *p *< .05. Additional tables were prepared using Excel 2002 (Microsoft Corporation, USA).

## Results

**Figure 3:**
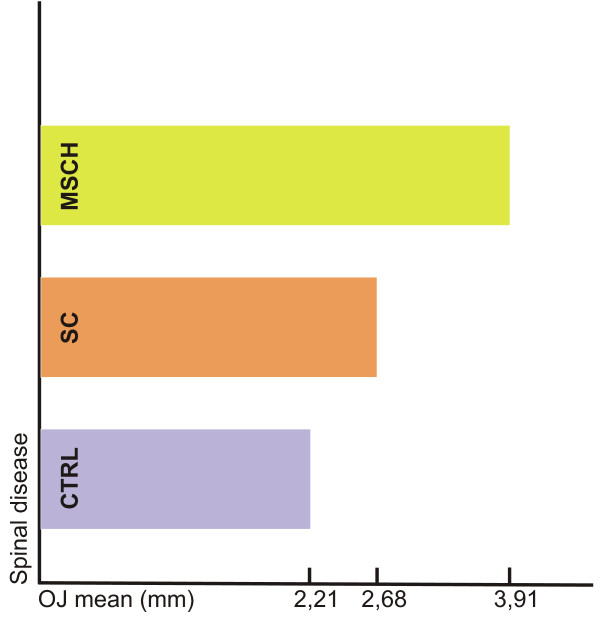
**Means of the overjet values**.

The sagittal abnormalities in the posterior region were examined by studying the molar relations. The unilateral and bilateral neutral occlusions, the distal occlusions and the mesial occlusions were registered separately [Table 1]. When analyzing the sagittal deviations in the molar region the incidence rate of the bilateral deviation being present as a sign of symmetry as well as of the unilateral deviation relating to the asymmetry has significant importance. The bilateral normocclusions were present in the CTRL group in the greatest proportion (64.68%) and the lowest (47.74%) in the MSCH group. In the case of maloccusions the frequency of the bilateral distal occlusion was twice higher in the MSCH group than the registered values of the SC group. Unilateral deviation was characteristic for almost one-third of both patient groups, while in the CTRL group the incidence rate was hardly 8.82%. In the patient groups the unilateral deviations were exclusively distal occlusions, while in the CTRL group the unilateral distal and mesial occlusions were present in the same proportion. The sagittal deviations in the frontal region were recorded by measuring the overjet of the incisors. The two examined groups possessed higher mean values compared to the determined mean value (2.21 mm) of the CTRL group. The mean values in the MSCH group were significantly higher than those of the SC group [Figure 3]. Having examined the repartition within the groups, the groups with severe spinal disease were found to be characterized by essentially higher deviations [Table 2]. Apart from the normal overjet, the cases representing an extreme overjet of ≥ 6 mm and a frontal crossbite were recorded separately. In the patient groups the frontal crossbite was not registered. Examining the values of the extreme overjet, we found that the significantly higher incidence rate and the average occurrence are characteristic for the MSCH group.

**Table 1:** Frequency of the sagittal occlusal anomalies on the posterior region.

Parameters		Scheuermann's disease group	Scoliosis group	Control group
Normal molar occlusion (Angle Cl.I) frequency(%)	unilateral	30.43	28.56	16.17
	
	bilateral	47.74	57.12	64.68

Distal molar occlusion (Angle Cl.II) frequency(%)	unilateral	30.43	28.56	8.82
	
	bilateral	21.73	10.07	16.17

Mesial molar occlusion (Angle Cl.III) frequency(%)	unilateral	~	~	7.35
	
	bilateral	~	3.57	2.94

**Table 2:** Comparison of the occlusal characteristics of the frontal region (Student's *t*-test).

Parameters	Scheuermann's disease group		Scoliosis group		Control group	*p*
	severe type	moderate type	severe type	moderate type		Student *t*-test
	4.27 ± 3.973					0.001**
Overjet		3.25 ± 2.188			2.21 ± 1.201	0.040*
mean ± SD (mm)			2.74 ± 1.851			0.143
				2.55 ± 1.509		0.438

	4.27 ± 3.150					0.039*
Overbite		2.75 ± 3.195			3.10 ± 1.585	0.601
mean ± SD (mm)			2.58 ± 2.168			0.245
				2.78 ± 1.715		0.568

	1.43 ± 0.724					0.125
Midline deviation		1.67 ± 1.061			1.47 ± 0.898	0.347
mean ± SD (mm)			2.08 ± 1.121			0.049*
				1.76 ± 0.972		0.597

* – significant

** – highly significant

Among the sagittal and skeletal parameters, the mean values of both patient groups can be found within the deviation range determined by Ricketts – both in respect of the maxillary depth and the ramus position. In view of the maxillary depth the mean values are higher than the normal ones (90 ± 3°), while in the case of ramus position they were lower (76 ± 3°) [Table 3].

**Table 3:** Cephalometric measurements in Scheuermann's disease and scoliosis group.

Direction of the measurement	Parameters	Scheuermann's disease group				Scoliosis group			
		Mean	SD	Min.	Max.	Mean	SD	Min.	Max.
Sagittal	Maxillary depth (MD) Norm:90.0 ± 3°	91.31	3.87	80.80	100.90	91.83	3.15	85.80	97.70
	
	Ramus position (RP) Norm:76.0 ± 3°	73.80	4.98	59.00	82.30	74.84	2.91	68.00	82.90

Vertical	Facial axis (FA) Norm:90.0 ± 3°	86.31	3.62	78.20	91.80	86.50	4.92	78.40	100.20
	
	Lower facial height (LFH) Norm:47.0 ± 4°	39.50	4.17	30.50	50.10	39.35	5.22	27.70	50.30
	
	Mandibular plane angle (MP) Norm:27.2 ± 4.5°	22.97	5.80	14.30	33.40	21.01	5.62	10.10	30.80

**Figure 4:**
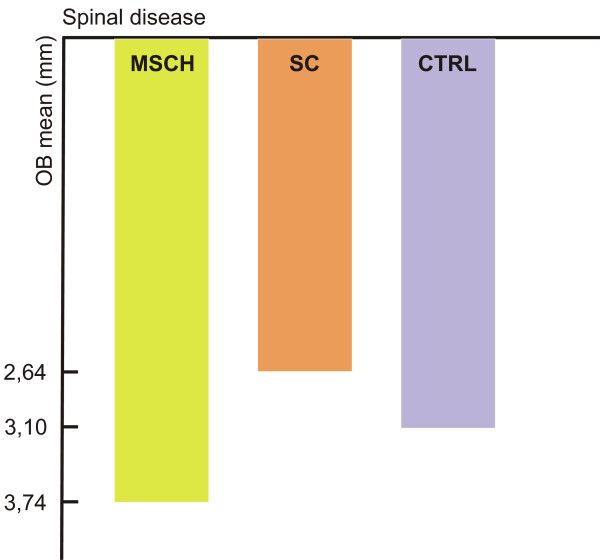
**Means of the ovebite values**.

Examining the data of the vertical abnormalities, there were no detectable posterior open bite cases; in order to determine the vertical anomalies of the frontal region the registered data of the normal overbite, the deep bite of ≥ 5 mm, and the open bite of ≥ 0 mm were used. The MSCH group was characterized by higher, while the SC group by lower overbite mean values compared to the determined mean values (3.10 mm) of the CTRL group [Figure 4]. Having examined the repartition within the groups, those with severe spinal disease were characterized by greater deviations in the MSCH group only [Table 2]. In the MSCH group, the frequency of the deep bite cases was higher, while the majority of the frontal open bite cases were registered in the SC group. In the case of the CTRL group the high incidence rate of the normal overbite and the lower rate of the deep bite as well as the lack of the open bite cases are striking.

The mean value of the three examined skeletal parameters with a vertical direction was lower than the normal values according to Ricketts. The facial axis values of the MSCH group were lower than the ones of SC group; in both patient groups, the measured values differentiated from the normal values concomitantly to the severity of the orthopedic malformation (90 ± 3°). This tendency was not characteristic in the case of values related to the lower facial height and the mandibular plane. In both cases, the values of the SC group showed a higher deviation from the normal values – lower facial height (47.0 ± 4°), mandibular plane (27.2 ± 4.5°) [Table 3].

The study of the occlusal anomalies characteristic to the studied groups was finalized with the analysis of the transversal relations. One child in the MSCH group and three children in the SC group were registered with a unilateral crossbite and there were 1-1 bilateral crossbites in both groups. In the CTRL group two bilateral crossbite cases matched three unilateral crossbite cases. The evaluation of the records of the frontal region gave a better picture. Comparable to the upper midline's deviation, the lower midline's deviation from the facial midline was more frequent in the MSCH group than in the SC group. The higher number of midline deviations was characteristic for the MSCH group; however the SC group was found to have higher deviation mean values. Having examined the repartition within the groups the midline deviation cases occurred three times more in the groups with severe type of spinal disease than in the group with moderate type of spinal disease. Regarding the degree of the deviation the severe type of SC group was characterized by significantly higher values compared to the CTRL group [Table 2]. Beside a similar manifestation frequency the CTRL group had lower deviation mean values in contrast to the patient groups.

The evaluation of the space anomalies in the jaws included the recording of the crowding and spacing in the upper and lower frontal regions. With almost equivalent crowding and upper spacing values, the occurrence of the spacing in the lower region showed a significant deviation, being lower in the CTRL group.

**Figure 5:**
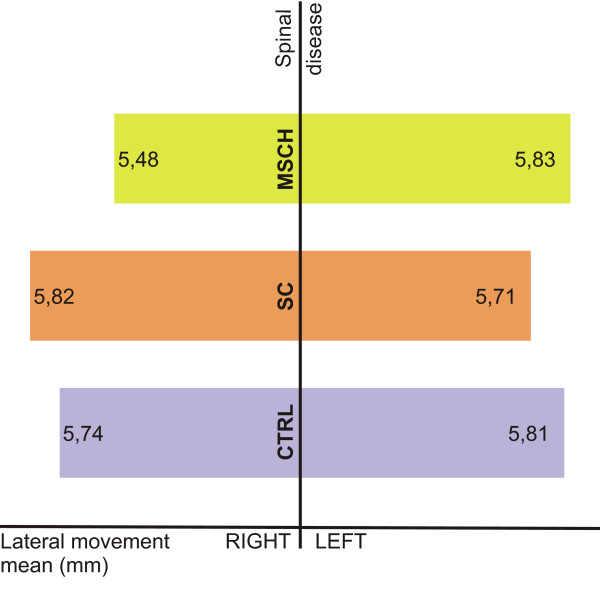
**Lateral movement ranges**.

The clinical examination of the TMJ at almost a quarter of the SC group revealed a pathological symptom as opposed to group MSCH where only 4.34% had an abnormality. Some pathological symptoms were found in a relatively low proportion (7.35%) in the CTRL group. The study of the mandibular lateral movement showed three significantly different movement ranges in the spinal-disorder groups and the control group [Figure 5]. Only half of the patients in the SC group were able to make the same range of bilateral movement, while in the MSCH group, 60.86% of the patients could. On the contrary this rate is 82.32% in the CTRL group.

In the course of the X-ray analyses, among the values (ramal height/RH/ and condylar plus ramal height/CH+RH/) measured on the similar sides of two orthopedic groups there were significant differences. The mean values of the MSCH group measured on both the left and right ramuses were higher than those of the SC group [Table 4]. The mean values of the asymmetry indices related to the mentioned lengths were similarly higher in the MSCH group (2.92 for RH and 2.27 for CH+RH), compared with the SC group (2.80 for RH and 1.93 for CH+RH). When comparing the sub-groups formed on the basis of the severity of the orthopedic deformation, higher asymmetry indices matched more severe deformations, for all the three lengths [Table 5].

**Table 4:** Comparison of the asymmetry indicators characteristic to the examined orthopedic patient groups (Student's *t*-test).

Parameters		Scheuermann's disease group		Scoliosis group		*p*
		mean	SD	mean	SD	Student *t*-test
Condylar height	Left	5.47	2.040	6.27	1.881	0.154 (NS)
(mm)	Right	5.97	2.016	6.10	1.537	0.799 (NS)

Ramus height	Left	54.12	5.281	49.74	4.681	0.003*
(mm)	Right	52.10	5.761	49.03	4.460	0.036*

Condylar+ramus height	Left	59.49	5.428	55.85	4.832	0.015*
(mm)	Right	58.07	5.565	55.12	4.858	0.049*

* – significant

NS – non significant

**Table 5:** Asymmetry indexes of the mandible in different orthopedic groups.

Asymmetry index	Scheuermann's disease group		Scoliosis group	
[(R-L)/(R+L)]x100	severe type	moderate type	severe type	moderate type
Condylar index mean ± SD	12.02 ± 11.32	11.06 ± 17.02	12.45 ± 8.53	12.24 ± 10.79

Ramal index mean ± SD	3.16 ± 2.18	2.47 ± 5.35	3.32 ± 3.18	1.72 ± 0.89

Condylar+ramal index mean ± SD	2.66 ± 2.25	1.54 ± 2.44	2.58 ± 2.41	0.56 ± 0.47

## Discussion

Numerous studies discuss the relationship between the multiple postural disorders and spinal illnesses causing these disorders and the dental complex. Among the examined spinal disorders, idiopathic scoliosis characterized by the pathologic curvature occurring in the frontal plane is relatively frequent, while the Scheuermann's disease with the pathological curvature in the sagittal plane is uncommon [5]. In the reviewed studies, the pathological postures correlating with the dominant kyphotic curvature are often encountered, however, the Scheuermann's kyphosis is not only evident in the case of the increased curvature values but also in the case of degenerative vertebral changes of the affected spinal section which cause the posture abnormality to become permanent [21,22]. The statistically high incidence values, the advancement of the disease over time and the pathological curvature that is normal for the scoliotic curvature were the reasons that the children with Scheuermann's disease participated in this epidemiological study. There are only few articles which describe the orthodontic examination as an opportunity for the early detection of spinal disorders or which emphasize the necessity of early orthodontic check-ups for children with diagnosed spinal disorders, highlighting the application of minimal-invasive methods of screening the affected population [4,9]. For these practical reasons, this study is also based on these methods.

Based on the previous results, a dominancy of the dentofacial asymmetry in the SC group, and sagittal and vertical alterations caused by the forward-tilted head posture and an increase in the numbers of the TMJ abnormalities in MSCH group were expected [1,3,5].

After the evaluation, in most cases our values were similar to those in the studies; however, we found some exceptions.

The frequency of the asymmetric deviations of posterior region occurring in the sagittal dimension was similar in the groups with spinal deformities and this value was double than the one registered in the CTRL group. The frequency of the symmetric normocclusions was the greatest in the CTRL group while the incidence values of malocclusions were the highest in the MSCH group. The frontal crossbites characterizing mostly the patients with scoliosis were not registered in our patient groups [9].

An examination of the incisor relation proved the previously found correlation between the alterations in the sagittal plane and the pathological kyphotic curvature; just like the extreme overjet, the deep bite was significantly more frequent in patients affected by Scheuermann's disease, in contrast to the SC or the CTRL group.

On the basis of the evaluation of cephalograms of the groups with spinal disorders, a slightly protrusive maxilla and a slightly retrusive mandibula are characteristic for a skeletal deep bite which is more pronounced in the SC group than in the MSCH group.

The quantity of lateral crossbites was minimal in both examined groups; however the transversal deviations of the frontal region could be evaluated. The incidence indicators of the dentofacial asymmetry characterizing the patients with scoliosis were exceeded by the values of the MSCH group in respect of the deviation of the upper and lower midline in relation to the facial midline as well as the midline deviation. The mean asset of the midline deviation was significantly higher in the scoliotic group, of which importance is increased by the lower mean value typical for the CTRL group, this value being even lower than of the MSCH group.

In literature, the opinions on the correlation between the poor head posture and alterations of the TMJ differ [4]. Some authors favor the theory that in case of the forward-tilted head position, the displaced centre of gravity can be a risk factor causing the development of the TMJ dysfunctions, others say that a laterally tilted head posture favors the mandible deviation loading the articulation asymmetrically [2,11]. The latter hypothesis seems to be proved by the numerous pathological symptoms of the TMJ registered among scoliotic patients, together with the significant number of asymmetrical values found in respect of the lateral movements. Despite the significant difference among them, the functional asymmetry index of both groups is twice greater than the values measured in the control group. The panoramic X-ray analyses show highest facial asymmetry indicators in case of the MSCH group. As a result of this the adequate asymmetry index comes from the comparison done after the condylar, ramal and condylar plus ramal height measurements performed on the two mandibular ramuses. As opposed to the condylar index both the ramal and the condyle plus ramal indices were higher in the MSCH group and therefore showed a greater asymmetry. The already mentioned asymmetry indicators are collaterally changing with the severity of the orthopedic deviations.

The difficulty of this range of subjects is shown by the concordant or the contrary results of this study compared to the ones previously found in the literature studying the etiological roles of the spinal deformities with unclear origins in the development of the craniofacial deformities [23].

## Conclusion

The data of this epidemiological study prove and partially complete the results of numerous studies in literature reporting a high number of dentofacial anomalies in children with various spinal diseases.

In both groups with spinal disorders there were differences shown between the size and the frequency of the occurrence of several deviations. The observed more frequent and more severe dentofacial deviation in the MSCH groups draws the attention to the necessity of the early examination of this less examined patient group from an orthodontics point of view.

## Competing interests

The authors declare that they have no competing interests.

## Authors' contributions

ES carried out the study design and initiated the study, carried out the cephalometric analysis, participated in the data collection and drafted the manuscript. CL participated in the idea for this study, data collection and manuscript drafting. AV participated in the design of the study, performed the coordination of the study and helped to perform the cephalometric analysis and to draft the manuscript. All authors read and approved the final manuscript.

## Pre-publication history

The pre-publication history for this paper can be accessed here:


